# Characterization of natural killer and T cells in bronchoalveolar lavage and peripheral blood of sarcoidosis patients

**DOI:** 10.3389/fimmu.2022.1080556

**Published:** 2023-01-04

**Authors:** Laura Bergantini, Miriana d’Alessandro, Genny Del Zotto, Emanuela Marcenaro, Elena Bargagli

**Affiliations:** ^1^ Respiratory Diseases Unit, Department of Medical Science, Surgery and Neurosciences, University of Siena, Siena, Italy; ^2^ Department of Research and Diagnostics, IRCCS Istituto Giannina Gaslini, Genova, Italy; ^3^ Dipartimento di Medicina Sperimentale (DIMES), Università degli Studi di Genova, Genova, Italy; ^4^ IRCCS Ospedale Policlinico San Martino, Genova, Italy

**Keywords:** sarcoidosis, bronchoalveolar lavage, interstitial lung diseases (ILD), natural killer (NK), T cells

## Abstract

The characterization of frequency and phenotypes of natural killer (NK) cells and T cells in BAL and peripheral blood of patients with sarcoidosis was evaluated, to discriminate the differential status of these cells in these two compartments. The analysis revealed that CD56^bright^CD16^neg^ resulted higher in BAL than PB of sarcoidosis and healthy subjects, while CD56^dim^CD16^+^ showed a different proportion between BAL and PB of both Sarcoidosis patients and HC. Moreover, in comparison with autologous PB, BAL was characterized by a higher expression of activated NK cell markers NKp44, CD69 and CD25. Significantly increased levels of PD-1^+^ NK cells in the BAL of patients were detected. Regarding the maturation of CD4 and CD8, an increase of Effector Memory T cells (T_EM_) was reported in BAL compared to PB. A better characterization of NK and T cells may lead to an improvement of the pathogenetic mechanisms in sarcoidosis.

## Introduction

Among Interstitial lung diseases of unknown origin, Sarcoidosis is classified as a chronic multisystemic disease that mainly involves the lung of adults and rarely in children (aged 13–15 years), with several clinical presentations and prognosis, characterized by non-necrotizing granulomatous inflammation (1–3).

The pathogenesis of sarcoidosis is already not fully understood due to the heterogeneity of its clinical characteristics and the unpredictable outcome that can be asymptomatic or can evolve into fibrosis with an irreversible process (4). Granulomas comprise several cellular lineages belonging to both innate and adaptive immune responses (5). Among these cell subsets, macrophages that differentiate into epithelioid cells play a key role in the formation and development of granuloma together with CD4^+^ T helper cells that are interspersed within the granuloma, while other cells such as CD8^+^ T cells surround the periphery (6, 7). CD4^+^ and CD8^+^ T cells characterize granuloma, however, only few studies characterized their functions and subsets at the periphery and into the lung (8, 9).

Bronchoalveolar lavage (BAL) is considered a standard diagnostic procedure in patients with suspicion of interstitial lung diseases (ILD) (10). It involves different cells from the lower respiratory tract, mainly represented by macrophages, lymphocytes, eosinophils, and neutrophils (11). Lymphocytes present in the interstitium of the lung represent the most easily accessible lymphocytes of the human lung (about 5% of the total circulating lymphocyte pool in humans) (11). By clinical data and chest X-ray, the presence of elevated lymphocytes (more than 15%) and CD4/CD8 ratio >3.5 support diagnosis of pulmonary sarcoidosis (12).

The knowledge regards Natural killers (NK) with the other Innate lymphoid cells (ILCs) was recently improved (13, 14). The understanding of NK cell biology has enhanced in terms of maturation, diversity, and adaptive capacities (15).

NK cells provide a first line of defense against infection and cancer (16). They express both inhibitory and activatory receptors. Among inhibitory receptors, killer Ig-like receptors (KIRs), and the CD94/NKG2A heterodimer, recognize major histocompatibility complex (MHC) class I molecules (17). Immune checkpoint PD-1 also belongs to the inhibitory receptors expressed of NK cell surfaces. PD-has recently described on a subset of peripheral blood (PB) NK cells from healthy HCMV^+^ individuals and NK cells from tumor patients (18–21).

When target cells lack expression of MHC-I molecules, NK cells start their activation with the expression of the natural cytotoxicity receptors (NCRs), i.e. NKp30, NKp44, and NKp46, NKG2D, DNAM-1 and NKG2C (the activating counterpart of NKG2A) (16, 18).

In the last years, the number of studies on NK cell features in the lung increased, showing that the lung contains a high reservoir of NK cells (15). The distribution of the various NK cell populations is similar to that of peripheral blood, with a majority of the more mature NK cells (CD56^dim^CD16^+^) and a minority of the immature CD56^bright^CD16^neg^ NK cells (22, 23).

Only a few works investigated NK cells in Sarcoidosis, and they were mainly focalized on their percentages at a peripheral and alveolar level in comparison with other ILDs for differential diagnosis (1, 24).

In the present study, we analyzed the frequency of NK and T cells and the expression of different NK and T surface markers in BAL and PB samples from sarcoidosis patients, to discriminate the differential status of NK and T cells in these two compartments.

## Materials and methods

### Study population

BALF and PB cells for each subject were obtained from 13 sarcoidosis patients (mean age 52 ± 14 years). The final diagnosis was performed by a multidisciplinary team at Siena University Hospital, following international criteria.

PB samples from a group of Healthy controls (HCs) were collected. They had no history of autoimmune, cancer, or other relevant diseases that can alter immunologic pathways. All the available variables of HCs were recorded in an electronic database.

The most relevant clinical characteristics are reported in **Table 1**. At the moment of time sampling, patients were not undergoing any treatments. All subjects gave their informed consent, and the study was approved by the local ethics committee (markerlung 17431).

**Table 1:** Demographic, immunologic and functional data of the cohort.

Subjects (n)	13
**Male/female**	3/10
**Age (year)**	52 ± 14
**Ex Smoker/never smoker (*n*)**	6/7
Chest X-ray stages (*n*)	
0	4
I	0
II	6
III, IV	3
Lesions other than lung (*n*)	
Heart	1
Skin	3
Eye	1
Brain	1
Liver	1
BALF cell count (mean ± SD)	
Cellular concentration (×10^6^ cells)	5.8 ± 2.3
Cell/ml (x10^3^)	96.4 ± 36.5
% of macrophages (%)	77 ± 16
% of lymphocytes (%)	19 ± 15
% of neutrophils (%)	3.3 ± 3.7
% of eosinphils (%)	0.4 ± 0.65
Peripheral cell count (mean ± SD)	
% of monocytes (%)	10.4 ± 2.5
% of lymphocytes (%)	24.2 ± 8.3
% of neutrophils (%)	61.3 ± 8.2
% of eosinphils (%)	3.5 ± 1.7
Biomarkers (mean ± SD)	
ACE (U/l)	62 ± 21
Lysozyme (mg/l)	5.3 ± 1.6
Pulmonary function tests (mean ± SD)	
FEV1%	92.7 ± 17.2
FEV1 ml	2383 ± 640
FVC %	94.4 ± 15
FVC ml	2998 ± 848
DLco (%)	71 ± 13

### BAL procedure and handling of cells

BAL and PBMC collection were performed in the laboratory of the Respiratory Diseases Unit, Siena University Hospital (Italy) from January 2019 to December 2020.

BAL was performed as previously described (25). BAL was filtered through sterile gauze. Cytocentrifuge smear was obtained for differential cell count with a Fast Quick - May Grunwald-Giemsa rapid (cat. Nr. 010253, DiaPath, Italy); Remaining cells were centrifuged at 406x*g* for 10 min at 4°C and resuspended in RPMI 1640 medium (Gibco, Paisley, UK). BALF cells were counted and trypan blue exclusion criteria were used for determining cell viability.

PB samples were drawn into a tube containing EDTA anticoagulant (BD Vacutainer^®^ EDTA tubes, BD Biosciences, CA, USA) and processed within eight hours. PBMC was obtained by gradient centrifugation separation (Ficoll Histopaque^®^-1077, Sigma-Aldrich). Cells obtained from BAL and PB were washed twice, resuspended in 80% RPMI1640, 10% FBS, and 10% Dimethyl sulfoxide (DMSO) at 2x10^6^ cells per vial, and stored in liquid nitrogen until analysis.

### Lung function tests

The following lung function parameters were recorded following standards international recommendation using a Jaeger body plethysmograph with corrections for temperature and barometric pressure. Forced vital capacity (FVC), forced expiratory volume in the first second (FEV1) and diffuse lung carbon monoxide (DLco) were performed and collected as volume (ml) and percentages of predicted values.

### Flow cytometry

All mAbs used in flow cytometry for the detection of surface markers are described in **Supplementary Table 1**. For multiparametric flow cytometric analysis, a standard staining protocol for extracellular markers was used (16). Cells were washed with Wash buffer (HBSS–/– with 2% of FBS), and incubated with antibodies mixed for 30 minutes in the dark at RT. Samples were detected using BD FACS Canto II (BD Biosciences). Titration experiments were defined for determining the optimal concentration. Fluorescence minus one (FMO) controls were used to determine accurate cytofluorimetric analysis following guidelines (26). For the analysis of cells, the total NK cell population was identified based on FSC vs SSC and negative for CD3, CD14, and CD19. CD56 was plotted against CD16 to obtain immature (CD56^bright^CD16^neg^) and mature (CD56^dim/neg^CD16^+^) phenotypes of NK cells. On the CD56^dim/neg^CD16^+^ population a series receptor was evaluated, including NKG2A, NKG2C, CD57, KIR, PD-1, CD25, CD69 and NKp44. For the detection of T Cell maturation, a panel including anti-CD3 APC-Cy7, CD4 FITC, CD62L PE, CD8 Vioblue, and CD45RA PE-Vio770 was used.

### Statistical analysis

Means and standard deviations (M ± SD) or medians and quartiles (25th and 75th percentiles) for continuous variables were used. A one-way ANOVA non-parametric test (Kruskal–Wallis test) and Dunn test were performed for the comparison of more than 2 groups. To identify the normal distribution of the variables, the Shapiro–Wilk test was applied. The Chi-squared test was used for categorical variables. Statistical analysis and graphic representation of data were performed by GraphPad Prism 9.0 software (Graphpad Holdings, LLC, San Diego, CA, USA).

A p-value of less than 0.05 was considered statistically significant.

Supervised principal component analysis (PCA) was employed to reduce the dimensionality of data hyperspace and for clusterization of the samples based on their cellular subsets.

For the multivariate analysis, the % of differential surface markers in the overall cohort was used to perform a supervised heatmap analysis; this analysis visualizes the percentages of the differential cellular markers in each enrolled patient. Clusterization was performed based on Spearman rank correlation and K means. The above analyses and corresponding figures were obtained using MORPHEUS (https://software.broadinstitute.org/morpheus/) and ClustVis (http://biit.cs.ut.ee/clustvis) software.

## Results

### Study population

No statistically significant differences were reported in terms of Sex distribution, age, and smoking habits for HC when compared with sarcoidosis patients. Demographic data (including sex, age, and smoking habits) of sarcoidosis patients are reported in **Table 1**. As expected, patients were young, prevalently female who had never smoked. At the chest X-ray, three patients report stage III or IV, four patients stage 0, and four patients stage II. Regarding BAL cell count, an increased percentage of lymphocytes was reported, while biomarkers and PFTs values were unaltered.

### NK cell analysis of PB and BAL of patients affected by sarcoidosis

**Figure 1:**
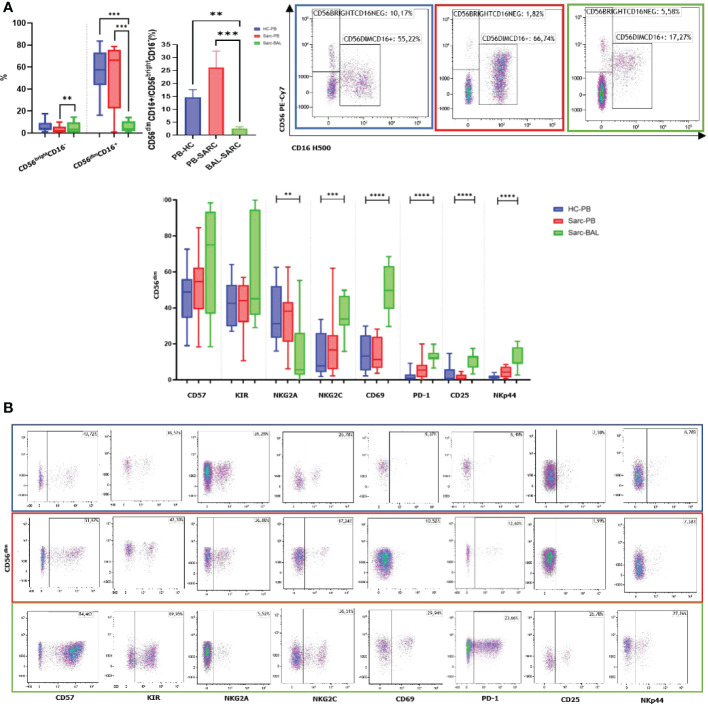
**(A)** Histograms and flow cytometric dot plot of CD56^bright^CD16^dim^ and CD56^dim/neg^CD16^+^ cell subsets in BAL of sarcoidosis patients and PB of sarcoidosis patients and healthy controls. **(B)** Histograms and flow cytometric dot plot of CD56^dim/neg^CD16^+^ cell subsets expressing CD57, KIR, NKG2A, NKG2C, CD69, PD-1, CD25, and NKp44 in BAL of sarcoidosis patients and PB of sarcoidosis patients and healthy controls. **p<0,01 ***p<0,001 ****p<0,0001.

As above mentioned, we analyzed a wide number of surface markers on peripheral blood (Sarc-PB) and BAL fluid (Sarc-BAL) NK cells of the selected patients. The results were compared with the peripheral blood of healthy controls (HC-PB). CD56^bright^CD16^neg^ showed an increased level in BAL than PB of sarcoidosis and healthy subjects. CD56^dim/neg^CD16^+^ at the same time showed a different proportion between BAL and PB of both Sarcoidosis patients and HC (**Figure 1A**). Moreover, from the analysis of CD56^dim/neg^/CD56^bright^ ratio, BAL samples reported significantly lower values of the ratio than PB of HC and Sarcoidosis (2,5 ± 2,4 Sarc-BAL, 26,1 ± 22,9 Sarc-PB, and 14,5 ± 10,6 HC-PB; p=0,0003) (**Figure 1A**).

In comparison with autologous PB, BAL was characterized by a higher expression of the activation NK cell markers NKp44, CD69, and CD25. In addition, NKG2A was decreased, and its activating counterpart (NKG2C) was increased (**Figure 1B**).

The levels of markers characterizing terminally differentiated NK cells, such as CD57 and KIRs, were higher in BAL than in the peripheral blood of both patients and HC (**Figure 1B**).

The inhibitory checkpoint PD-1 showed a similar trend, as it was negative on almost all HD-NK cells, highly positive on a small percentage of Sarc-PB while a highly expressed on a large fraction of BAL-NKs.

### T-cell analysis of peripheral blood and Bronchoalveolar lavage of patients affected by sarcoidosis

Due to the crucial immune-pathogenetic role of lymphocytes in granuloma formation of sarcoidosis, analysis of T cell subsets resulted in fundamental to improve the knowledge of pathogenic mechanisms of this disorder.

**Figure 2:**
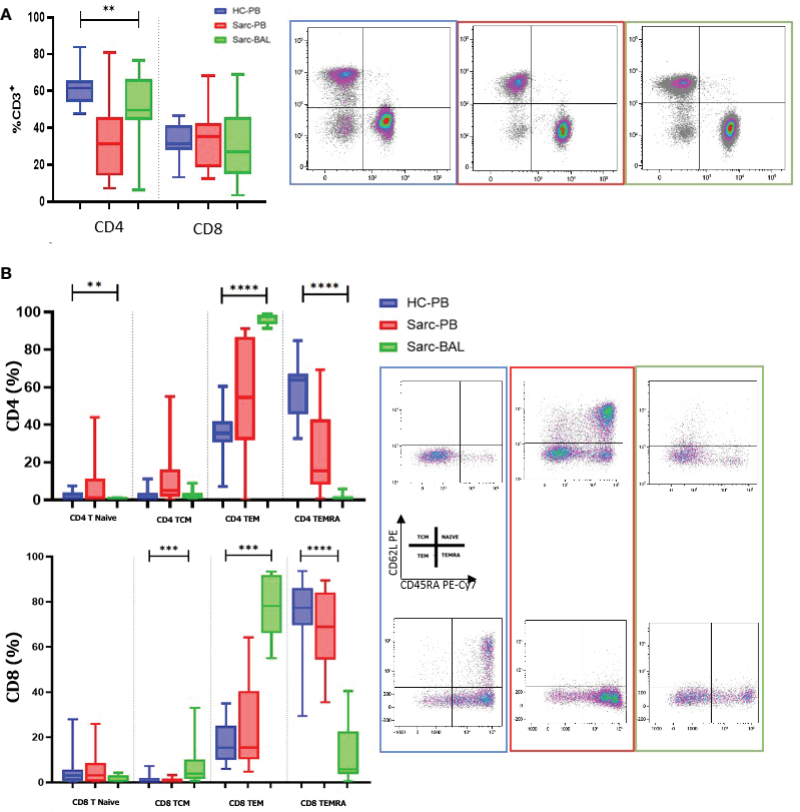
**(A)** Histograms and flow cytometric dot plot of CD4 and CD8 T cell subsets in BAL of sarcoidosis patients and PB of sarcoidosis patients and healthy controls. **(B)** Histograms and flow cytometric dot plot of T Naive, TCM, TEM, and TEMRA of BAL of sarcoidosis patients and in PB of sarcoidosis patients and healthy controls. **p<0,01 ***p<0,001 ****p<0,0001.

Interestingly, as expected, a predominance of CD4^+^ T cells was reported in BAL compared to patients’ PB, typical of the recruitment of helper T cells into the granuloma. Regarding the maturation of CD4 and CD8, an increase of Effector Memory T cells (T_EM_) was reported in BAL compared to PB. On the other hand, CD4 and CD8 T_EM_RA showed decreased percentages in BAL than PB. A decreased level of CD4^+^ naïve T cells was reported. CD8^+^ naive T cells showed the same trend however without reaching significance. Concerning Central Memory T lymphocytes (T_CM_), only CD8 showed an increased level in BAL than PB (**Figures 2A, B**).

### PCA and Heatmap analysis revealed the same biological behavior among groups

Based on the flow cytometry data, we performed a PCA analysis on all the different cell subgroups detected on the NK and T cell surfaces in BAL and PB. The PCA plot shows that samples with the same biological behavior clustered together, corroborating that the differential cell subsets were characteristic for each condition.

**Figure 3:**
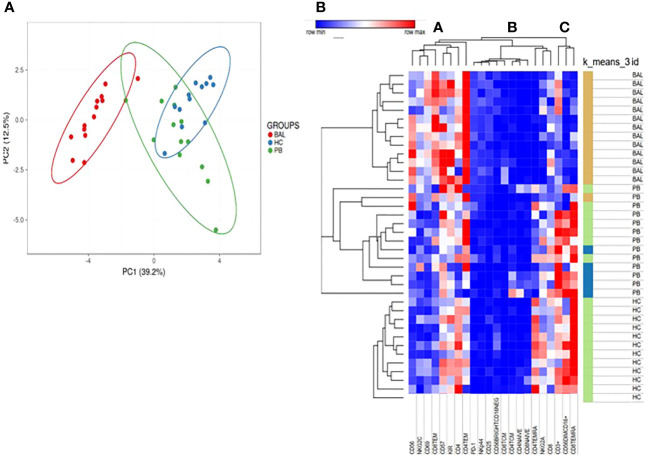
**(A)** For PCA analysis, Unit Variance Scaling is applied to rows; SVD is used to calculate principal components. X and Y axis show principal component 1 (PC1) and principal component 2 (PC2) that explain 39.2% and 12.5% of the total variance, respectively. Prediction ellipses are such that with probability 0.95. **(B)** Heatmap analysis performed on cell surface markers of NK and T cells in PB and BAL of sarcoidosis patients and PB of HC based on hierarchical clusterization based on spearman rank correlation. K means was also applied to detect clusters based on the expression of surface markers.

Furthermore, the PB of sarcoidosis patients clusters close to the PB of HC. On the other hand, the BAL samples were located on the opposite side of the PB samples (**Figure 3A**).

In particular, Unit Variance Scaling is applied to rows and SVD is used to calculate principal components. PC1 and PC2 explain 39.2% and 12.5% of the total variance, respectively (**Figure 3A**).

**Figure 3B** showed heatmap analysis performed on cell surface markers of NK and T cells in PB and BAL of sarcoidosis patients and PB of HC based on hierarchical clusterization based on spearman rank correlation. Similar to PCA analysis, the general trend separates samples into three principal groups as indicated by the dendrogram on the top of the matrix and indicated as A, B, and C.

Of note, group A is mainly composed of CD56 cell surface markers, including CD57, NKG2C, and KIR. The B group includes mainly subsets of T cells as T central memory and Naïve T cells.

K means was also applied to detect clusters based on the expression of surface markers (**Figure 3B**).

The analysis revealed an inverted trend in the expression of several surface markers on the BAL samples with respect to PB of both sarcoidosis and HC group.

## Discussion

In this study, an evaluation of different surface cell markers, phenotypically and functionally characterizing NK and T cells, was performed in the BAL and PB of sarcoidosis patients and HCs. Moreover, the lung microenvironment typical of patients affected by sarcoidosis was explored through the analysis of BAL cell subsets. These biological data play an important role in diagnosis and they provide interesting information on the cells in the interstitial space of the lung. From the clinical point of view, the selected patients can be considered representative of a typical sarcoidosis patients’ cohort in terms of age and gender distribution as well as of predominance of stage 2 at chest X-ray.

In multivariate analysis, a clear division of the three groups (Sarc-BA, Sarc-PB, and HC-PB) emerged. This result showed that the analyzed NK and T cell subsets greatly differentiate among the three groups, as clearly reported in **Figure 3**.

Different studies reported that, upon *in vitro* stimulation, there is an increase of IFN-γ and TNF-α produced by immature CD56^bright^ NK cells in BALF of sarcoidosis patients, and this may suggest the involvement of NK cells in granuloma formation (27, 28). Moreover, these studies seem to suggest that the more immature NK cells (CD56^bright^CD16^neg^) producing a large amount of Th1 cytokines (IFN-γ and TNF-α) may be involved in the pathogenesis of sarcoidosis (28, 29).

In line with the literature, we observed an increased fraction of the immature CD56^bright^ CD16^neg^ NK cell subset and a decrease of the more mature CD56^dim/neg^CD16^+^ NK phenotype in BAL of patients compared to their PB. Importantly, deep characterization of the CD56^dim/neg^CD16^+^ NK cell subset in BAL compared to autologous PB showed a large fraction of this more mature NK cell subset expressing KIR and a small percentage of NKG2A^+^ NK subpopulation. Furthermore, in BAL-NK cells were characterized by a high amount of CD57 (a marker of terminal differentiation) and NKG2C, the activating counterpart of NKG2A, generally upregulated during HCMV infection/reactivation. Unfortunately, our study lacks information on patients’ HCMV status. Moreover, CD56^dim/neg^CD16^+^ BAL-NK cells expressed activation markers, such as CD69 (which also represents a tissue-resident marker), NKp44, and CD25.

Regarding CD25, the soluble form “sCD25” was widely used as a serum marker of sarcoidosis active status. Recently it was demonstrated that, in the context of inflammation, CD56^dim^ NK cells expressing CD25 can be activated by IL-2-producing T cells during adaptive immune responses (30, 31). After stimulation with IL-2, NK cells can acquire NKp44, an activating NK cell receptor, involved in the triggering of NK cell cytotoxicity against target cells expressing the relative ligands. NKp44 has never been analyzed before in sarcoidosis patients and it could deserve further investigation.

It is interesting to note that we first described an overexpression of PD-1 in NK cells of BAL samples when compared to the PB of the same patient. In this regard, it has recently been shown that the expression of PD-1 induces an impairment of the function of NK cells towards the target cells expressing the relative ligands (PD-L1/2) thus demonstrating its role as an immune checkpoint also in NK cells (21). Upregulation of PD-1 was also present in PB CD4^+^ T cells of sarcoidosis patients (32, 33).

Braun et al. showed that spontaneous clinical resolution of sarcoidosis corresponds to a reduced percentage of PD-1^+^ CD4^+^ T cells, whereas clinical progression to an increase of PD-1^+^CD4^+^ T cells suggesting that the blockade of the PD-1 pathway may contribute to the restoration of CD4^+^ T-Cell Proliferative Capacity in Sarcoidosis patients (32). Moreover, in the same study, an increase in PD-1 levels in BAL compared to PB was also reported exactly as in our cohort of patients. In chronic beryllium diseases, another lung granulomatosis, PD-1 expression on CD4^+^ T cells directly correlated with the severity of T-cell alveolitis (34).

Although in sarcoidosis the exact role of PD-1 on NK cells was poorly investigated, in other granulomatosis of the lung it has been demonstrated that the PD-1 pathway impaired NK cell functions reducing IFN-γ production and lytic degranulation (35). Further investigation to unravel the role of PD-1 in controlling inflammation in sarcoidosis pathogenesis will be necessary.

In this study, we also evaluated the T cell compartment, in particular: naive, central memory, effector memory, and RA^+^ effector memory subsets of both CD4^+^ and CD8^+^ T cells.

In our study, BAL samples of sarcoidosis patients largely consisted of T _em_ lymphocytes, belonging both to the helper and the cytotoxic compartment T_EM_ cells represent an immediate defense, whereas T_CM_ cells support the response by proliferating in the secondary lymphoid organs and producing a supply of new effectors (35).

In many studies, it has been shown that lung resident T_EM_ cells can mediate early control of respiratory viral infections but they are inefficient at mediating recall responses in terms of proliferation and accumulation at inflammatory sites (36, 37). In other studies focused on lung malignancies, upregulation of both T_EM_ and T_CM_ was reported with a higher amount of cytokine released compared to T_EM_RA and T naïve, thus demonstrating their activity in the site of inflammation (38, 39).

In conclusion, in this study, a different NK cell subset distribution was observed at the site of inflammation compared to the PB of sarcoidosis patients (a higher proportion of CD56^bright^ as compared to CD56^dim/neg^ was observed in BAL). In addition, the more mature NK cell subset present in BAL is characterized by overexpression of activation markers, such as CD69, CD25, as well as NKp44, and a large fraction of fully mature NK cells, characterized by the NKG2A^-^, KIRs^+^ phenotype. Interestingly, these cells also express high levels of NKG2C and PD-1, as previously described in adult HCMV^+^ HC (21). The lack of prior research on specific aspects makes our research of interest and useful for further investigation. In this study, the characterization of NK and T- cell subsets in sarcoidosis revealed a distinct phenotype between the bloodstream and lung. Elevated levels of PD-1^+^ NK cells in the BAL of patients were observed. Other studies need to determine the functions of these cells. A deeper characterization of these cells can lead to a better understanding of the pathogenetic mechanisms of sarcoidosis.

## Data availability statement

The raw data supporting the conclusions of this article will be made available by the authors, without undue reservation.

## Ethics statement

The studies involving human participants were reviewed and approved by markerlung 17431. The patients/participants provided their written informed consent to participate in this study.

## Author contributions

LB and Md’A: conception and design of the study. GZ and EM: analysis of the data and their interpretation. LB: statistical analysis. LB and Md’A wrote the first draft of the manuscript. EB, EM, and GZ wrote sections of the manuscript. All authors contributed to the manuscript and approved the submitted version.
