# Supplementary material for: Characterization of natural killer and T cells in bronchoalveolar lavage and peripheral blood of sarcoidosis patients
Source: Front Immunol. 2023 Jan 4;13:1080556. doi: 10.3389/fimmu.2022.1080556 (PMC9846229; doi:10.3389/fimmu.2022.1080556)
Supplement: Supplementary file 1 [file Table_1.docx]

|  |  |  |  |  |  |
| --- | --- | --- | --- | --- | --- |
| **CD** | **Alternative name** | **Clone** | **Isotype** | **Fluorochrome** | **Company** |
| CD3 | T3, CD3ε | UCHT1 | IgG1 | APC-Cy7 | Biolegend |
| CD4 | T4, Leu3 | RPAT4 | IgG1 | FITC | Biolegend |
| CD8 | Leu-2, T8 | REA734 | IgG1 | VioBlue | Miltenyi |
| CD14 | Leu-M3, LPS-R | HCD14 | IgG1 | APC-Cy7 | Biolegend |
| CD16 | FCRγIII | 3G8 | IgG1 | BV510 | Biolegend |
| CD19 | B4 | HIB19 | IgG1 | APC-Cy7 | Biolegend |
| CD25 | IL-2Rα | BC96 | IgG1 | BV421 | Biolegend |
| CD45RA |  | REA 562 | IgG1 | PE-Vio770 | Miltenyi |
| CD57 | HNK-1 o Leu7 | TB03 | IgM | VioBlue | Miltenyi |
| CD56 | NCAM | HCD56 | IgG1 | PE-Cy7 | Biolegend |
| CD62L | L-selectin | DREG-56 | IgG1 | PE | Biolegend |
| CD69 | Leu23 | FN50 | IgG1 | BV421 | Miltenyi |
| CD158a | KIR2DL1 | HP-3E4 | IgM | FITC | Becton Dickinson |
| CD158b | KIR2DL2/DL3 | CH-L | IgG2b | FITC | Becton Dickinson |
| CD159a | NKG2a | Z199 | IgG2b | APC | Beckman Coulter |
| CD159c | NKG2C | REA 205 | IgG1 | PE | Miltenyi |
| CD336 | NKp44 | Z231 | IgGI | PE | Beckman Coulter |
| CD279 | PD1 | PD1.3.1.3 | IgG2b | PE | Miltenyi |

**Suppl. Tab. 1** Monoclonal antibodies used for the analysis of NK and T cell subsets.
